# Age-related alterations in efferent medial olivocochlear-outer hair cell and primary auditory ribbon synapses in CBA/J mice

**DOI:** 10.3389/fncel.2024.1412450

**Published:** 2024-06-26

**Authors:** Nele Marie Dörje, Liana Shvachiy, Fabian Kück, Tiago F. Outeiro, Nicola Strenzke, Dirk Beutner, Cristian Setz

**Affiliations:** ^1^University Medical Center Göttingen, Department of Otolaryngology-Head and Neck Surgery, InnerEarLab, Göttingen, Germany; ^2^University Medical Center Göttingen, Institute for Auditory Neuroscience, Göttingen, Germany; ^3^University Medical Center Göttingen, Department of Experimental Neurodegeneration, Center for Biostructural Imaging of Neurodegeneration, Göttingen, Germany; ^4^Institute of Physiology, Faculty of Medicine, University of Lisbon, Lisbon, Portugal; ^5^Cardiovascular Centre, University of Lisbon, Lisbon, Portugal; ^6^University Medical Center Göttingen, Department of Medical Statistics, Core Facility Medical Biometry and Statistical Bioinformatics, Göttingen, Germany; ^7^Max Planck Institute for Multidisciplinary Sciences, Göttingen, Germany; ^8^Translational and Clinical Research Institute, Faculty of Medical Sciences, Newcastle University, Newcastle upon Tyne, United Kingdom

**Keywords:** age-related hearing loss, cochlear efferent synapses, cochlear synaptopathy, MOC-OHC synapse, olivocochlear efferents, presbycusis

## Abstract

**Introduction:**

Hearing decline stands as the most prevalent single sensory deficit associated with the aging process. Giving compelling evidence suggesting a protective effect associated with the efferent auditory system, the goal of our study was to characterize the age-related changes in the number of efferent medial olivocochlear (MOC) synapses regulating outer hair cell (OHC) activity compared with the number of afferent inner hair cell ribbon synapses in CBA/J mice over their lifespan.

**Methods:**

Organs of Corti of 3-month-old CBA/J mice were compared with mice aged between 10 and 20 months, grouped at 2-month intervals. For each animal, one ear was used to characterize the synapses between the efferent MOC fibers and the outer hair cells (OHCs), while the contralateral ear was used to analyze the ribbon synapses between inner hair cells (IHCs) and type I afferent nerve fibers of spiral ganglion neurons (SGNs). Each cochlea was separated in apical, middle, and basal turns, respectively.

**Results:**

The first significant age-related decline in afferent IHC-SGN ribbon synapses was observed in the basal cochlear turn at 14 months, the middle turn at 16 months, and the apical turn at 18 months of age. In contrast, efferent MOC-OHC synapses in CBA/J mice exhibited a less pronounced loss due to aging which only became significant in the basal and middle turns of the cochlea by 20 months of age.

**Discussion:**

This study illustrates an age-related reduction on efferent MOC innervation of OHCs in CBA/J mice starting at 20 months of age. Our findings indicate that the morphological decline of efferent MOC-OHC synapses due to aging occurs notably later than the decline observed in afferent IHC-SGN ribbon synapses.

## Introduction

Hearing loss is the most prevalent sensory deficit in humans and the leading cause of years lived with disability for people aged 70 years and above (Haile et al., 2021). There has been substantial discourse surrounding the potential factors that underlie the clinical onset of age-related hearing deterioration, given its heterogeneous manifestation within the general population. It has been widely acknowledged, though, that cochlear aging is supported by a multifactorial process of intricate and multifaceted layers of complexity corresponding to distinct pathophysiological processes within the inner ear. The sensitivity of the peripheral auditory system is centrally modulated by efferent nerve fibers. Categorized into lateral and medial efferent auditory nerve fibers depending on the location of their cell bodies within the superior olivary complex, auditory efferent neurons selectively project to the organ of Corti within the cochlea. Neurons from the medial olivocochlear (MOC) pathway are myelinated and make contact to outer hair cells (OHCs), while lateral olivocochlear (LOC) efferents have thin unmyelinated axons and innervate the dendrites of afferent type I spiral ganglion neurons (SGNs) below inner hair cells (IHCs) (Warr and Guinan, 1979). Animal research indicates LOC efferents project to the ipsilateral cochlea (Robertson, 1985), while the majority of MOC fibers cross the midline and innervate the contralateral ear (Guinan et al., 1983).

Research has consistently linked the MOC efferent system to modulate OHC acoustic gain, improving the perception and differentiation of acoustic stimuli in noisy environments (Winslow and Sachs, 1988; Liberman and Guinan, 1998), including the ability to discriminate sound location (May et al., 2004; Andéol et al., 2011), as well as refining selective attention in acoustically complex circumstances (Delano et al., 2007; Terreros et al., 2016). Extensive evidence points to the significant influence of the MOC efferent pathway in protecting the cochlea against noise-induced damage (Kujawa and Liberman, 1997; Rajan, 2000; Taranda et al., 2009; Maison et al., 2013; Boero et al., 2018). Regarding aging, postmortem studies of human ears have indicated an age-associated reduction of MOC efferent projections (Liberman and Liberman, 2019), correlating with a functional decline of MOC activity with age (Parthasarathy, 2001; Kim et al., 2002; Jacobson et al., 2003; Konomi et al., 2014; Lisowska et al., 2014). While MOC efferents have been suggested to ameliorate age-related alterations in the cochlea at low and mid frequency ranges in mice (Liberman et al., 2014; Boero et al., 2020), there is limited information about how MOC efferent projections contacting OHCs might vary throughout the lifetime. Bringing together all the evidence that highlights an essential protective function of the MOC efferent auditory pathway, it becomes evident that there is a strong necessity to delve deeper into comprehending its role in the cochlear aging process.

The onset and progression of hearing loss in diverse mouse models as they age demonstrate significant heterogeneity, representing a substantial obstacle in the pursuit of advancing our translational comprehension of the cochlear aging process. Considering that the deterioration of auditory sensitivity with age displays a gradual progression in humans, accurately portraying the main physiological aspects of audiological decline linked to senescence is crucial for investigating its deterioration effectively. Given the distinct manifestations of age-related changes across different mouse strains (Zheng et al., 1999), CBA mice stand out as a well-documented mouse model which exhibit resilience against strain-associated early-onset age-related hearing loss (Sha et al., 2008; Ohlemiller et al., 2010; Sergeyenko et al., 2013; Kobrina et al., 2020). The CBA/J inbred mouse strain was selected for this study due to its resemblance to human presbycusis, as they do not show signs of hearing loss until later in life (Spongr et al., 1997; Sha et al., 2008; Ohlemiller et al., 2010). It has been strongly suggested that afferent cochlear synaptopathy, which impacts synapses between IHCs and type I afferent SGNs, is a primary factor in the age-related hearing decline observed in CBA mice (Kujawa and Liberman, 2009; Sergeyenko et al., 2013; Parthasarathy and Kujawa, 2018). Based on the observations of morphological age-related synaptic alterations in primary auditory synapses in this strain, and considering the points previously delineated, it remains pertinent to acquire additional knowledge regarding potential de-efferentation processes affecting synapses associated with myelinated efferent pathways, such as the MOC efferent system.

In this study, we performed a thorough age-related characterization of cochlear efferent and afferent synapses originating from myelinated auditory nerve fibers, involving 73 mice from the CBA/J strain. 60 CBA/J mice between 10 and 20 months were grouped based on bi-monthly intervals and compared with 13 counterparts aged 3 months. For each of the animals, both efferent synapses between MOC efferent nerve fibers and OHCs, as well as afferent synapses involving IHCs and type I afferent nerve endings from SGNs, were quantified based on their morphological integrity along the cochlear spiral at the apical, middle, and basal turns, followed by a comparative analysis based on age.

## Materials and methods

### Animals

Animal handling and experiments were carried out according to approved animal research protocols in accordance with the guidelines of the German Animal Welfare Act and authorized by the Animal Welfare Office of the State of Lower Saxony and the University Medical Center Göttingen, Germany. Mice were bred and housed in groups at the animal care facility of the University Medical Center Göttingen, Germany. Animals were kept at 23°C with *ad libitum* access to food and water following a regular mouse diet, a 12-h light–dark cycle, and a quiet, low-traffic vivarium. A total of 73 gender-mixed CBA/J mice (*n* = 73; 38 females and 35 males) ranging in age from 3 to 20 months were used for the experiments. 13 CBA/J mice (*n* = 13; 9 females and 4 males), all aged 3 months, were designated as the younger control group for comparative analysis. Sixty older CBA/J mice (*n* = 60; 29 females and 31 males), aged between 10 and 20 months, were divided into six age groups with a 2-month interval as follows: 10 months old (*n* = 9; 5 females and 4 males), 12 months old (*n* = 8; 5 females and 3 males), 14 months old (*n* = 9; 6 females and 3 males), 16 months old (*n* = 10; 5 females and 5 males), 18 months old (*n* = 13; 4 females and 9 males), and 20 months old (*n* = 11; 4 females and 7 males).

### Tissue extraction

The mice were humanely euthanized through CO_2_-inhalation. Prior to decapitation, mice were transcardially perfused with phosphate-buffered saline (PBS) followed by freshly prepared 4% formaldehyde in PBS. Both temporal bones were extracted and subsequent dissection was conducted in ice-cold PBS under a dissecting microscope. The otic capsule was carefully opened facing the helicotrema and along the round and oval windows. These areas were gently further exposed to facilitate fluid diffusion into the cochlea. The inner ears were subsequently immersed in freshly prepared ice-cold 4% formaldehyde in PBS and postfixed for 20 min while placed on ice. The left or right ear assignment was randomized before fixation. After three washing steps in PBS, the inner ears were placed in ethylenediaminetetraacetic acid solution with a concentration of 0.5 M and a pH of 8.0 at room temperature (RT) between 24 and 48 h for decalcification. After repeated washing steps in PBS, the organs of Corti were microdissected from the cochleae and segmented into basal, middle, and apical turns, while discarding the hook region during preparation. Each animal’s cochlea was randomly allocated for either efferent MOC-OHC synapse or afferent IHC-SGN synapse immunostaining.

### Immunohistochemistry

The individual apical, middle, and basal turns of the organs of Corti were transferred into pre-prepared shallow molds, each filled with PBS, within a 24-well plate covered with parafilm. Following this, the samples were permeabilized and blocked using goat serum dilution buffer (GSDB), containing normal goat serum at a dilution of 1:6, 450 mM NaCl, 20 mM phosphate buffer, and 3% Triton X-100. The samples were placed in GSDB for 2 h at RT, followed by an overnight incubation at 4°C in GSDB supplemented with the primary antibody. The primary antibodies utilized throughout this study are summarized in Table 1: guinea pig anti-Synapsin1/2 (Synaptic Systems, 1:400), rabbit anti-SK2 (Sigma-Aldrich, 1:200), rabbit anti-Ribeye A-Domain (Synaptic Systems, 1:200), chicken anti-Homer1 (Synaptic Systems, 1:200), mouse anti-MyosinVIIa (Developmental Studies Hybridoma Bank, 1:200). The anti-MyosinVIIa antibody (MYO7A 138–1) was obtained from the Developmental Studies Hybridoma Bank (DSHB), created by the NICHD of the NIH and maintained at the Department of Biology of The University of Iowa, IA, USA. The following day, the samples were washed with PBS and then incubated with the secondary antibody in GSDB at a 1:200 dilution for 1 h at RT. The following secondary antibodies were used: goat anti-mouse Alexa Fluor 488 (#A-11001, Invitrogen), goat anti-chicken Alexa Fluor 488 (#A-11039, Invitrogen), goat anti-guinea pig Alexa Fluor 568 (#A-11075, Invitrogen), goat anti-mouse STAR580 (#2–0002–005-1, Abberior), goat anti-rabbit STAR635P (#2–0012–007-2, Abberior). After three washes in PBS, the samples were mounted on glass slides using a Mowiol-based mounting medium and then stored at 4°C shielded from light.

**Table 1:** Antibody labeling.

Antibody	Immunogen	Source – Host	Identifier	Validation	Cochlear staining pattern
Anti-Homer1 (1:200)	Recombinant N-terminal half of human Homer1	Sysy* – chicken polyclonal	160–006, Lot# 1–9 RRID: AB_2631222	Validated by relative expression by Sysy* – widely used as post-synaptic marker (Jafari et al., 2021; Lloyd et al., 2023; Rueda-Carrasco et al., 2023)	Postsynaptic density (Michanski et al., 2019)
Anti-MyosinVIIa (1:200)	HIS-tagged synthetic peptide in the human MYO7A sequence (a.a. 927–1,203)	DSHB** – mouse monoclonal	MYO7A 138–1 RRID: AB_2282417	Immunohistochemistry, immunoblot/Western blot (Soni et al., 2005)	Hair cells (Majumder et al., 2017; Jeng et al., 2020; Kim and Ricci, 2022)
Anti-Ribeye (1:200)	Recombinant protein from rat Ribeye (a.a. 95–207)	Sysy* – rabbit polyclonal	92–103, Lot# 1–11 RRID: AB_2086775	Immunohistochemistry (Mesnard et al., 2022)	Hair cell ribbon synapses/pre-synapse (Carrott et al., 2016; Calvet et al., 2022)
Anti-SK2 (1:200)	Recombinant synthetic peptide from rat SK2 (a.a. 542–559)	Sigma-Aldrich – rabbit polyclonal	P0483 RRID: AB_260860	Immunohistochemistry, immunoblot, immuno-precipitation (Scholl et al., 2014)	OHCs – basolateral membrane, adult mice (Rüttiger et al., 2004)
Anti-Synapsin1/2 (1:400)	Synthetic peptide from rat Synapsin1 (a.a. 2–28)	Sysy* – guinea pig polyclonal	106–004, Lot# 1–25 RRID: AB_1106784	Validated by immunoblot / Western blot by Sysy* using Synapsin KO mouse tissue lysates	Olivocochlear efferent terminals (Hua et al., 2021; Chepurwar et al., 2023)

Sysy*, Synaptic Systems; DSHB**, Developmental Studies Hybridoma Bank.

### Confocal imaging

The imaging process was conducted using an Abberior Expert Line STED microscope (Abberior, Göttingen, Germany) based on an Olympus IX83 inverted microscope, equipped with excitation lasers at 488, 561, and 640 nm. Dissected cochlear samples, divided into apical, middle, and basal turns, were imaged with confocal microscopy using a 100 × 1.4 UPlanSApo oil immersion objective. Image stacks were captured with the Imspector Software v16.3 at a 15 μs dwell time and a pinhole set to 1.1. Confocal images were acquired with an xy pixel size of 80 nm by 80 nm covering a range of 15 to 30 OHCs and 10 to 20 IHCs per acquisition frame. Z-stack intervals ranged between 100 and 200 nm. Each z-stack was taken to span the entire vertical axis of the hair cells from cuticular plate to the synaptic pole, capturing all contacting MOC nerve terminals in the OHC region, and including all contacting type I afferent SGN nerve terminals in the IHC region. According to the physiological cochlear place-frequency map described for CBA/J mice (Müller et al., 2005), the chosen z-stack areas focused on the 6–8 kHz region in the apical turn, the 18–22 kHz region in the middle turn, and the 34–40 kHz region in the basal turn of the cochlea.

### Imaging analyses

The confocal images were processed for analyses utilizing the Fiji v2.9.0 open-source software (Schindelin et al., 2012) and Imaris v10.0 (Bitplane, Belfast, UK). For the apical, middle and basal turns, each integral efferent MOC-OHC or afferent IHC-SGN synapse was defined by the presence of aligned juxtaposed immunofluorescence spots of tagged pre-synaptic and post-synaptic proteins. An integral efferent MOC-OHC synapse was identified by the presence of fluorescence-tagged Synapsin1/2 within the pre-synaptic MOC efferent nerve terminal juxtaposed with the fluorescence-tagged SK2 protein channel located at the basolateral cell membrane of OHCs. Efferent MOC-OHC synapses were manually counted with support of the Analyze Particle plugin from Fiji. Afferent IHC-SGN synapses were visualized using three-dimensional renderings of each confocal z-stack using Imaris v10.0 (Bitplane). Integral afferent IHC-SGN synapses were defined as juxtaposed immunofluorescence spots at IHCs’ active zones, representing the immunostained pre-synaptic protein Ribeye and the postsynaptic protein Homer1, localized within the afferent nerve terminal bouton of the corresponding type I afferent SGN fiber. To mitigate subconscious bias in afferent synapse counting relative to the mouse age and the contralateral ear stained for MOC-OHC efferent synapses, the experimenter was blinded to the specific experimental condition. Afferent IHC-SGN ribbon synapses were manually identified with the assistance of the Spot Detection plugin in Imaris v10.0 (Bitplane). A ribbon synapse was considered morphologically intact based on direct visualization of congruently juxtaposed fluorescent spots representing pre-synaptic (Ribeye) and post-synaptic (Homer1) staining. IHC-SGN synapses were further categorized as modiolar or pillar based on their anatomical orientation on IHCs, with the longitudinal axis of IHCs serving as the primary plane of section. This allowed for the distinction between synapses oriented towards the modiolus and those oriented towards the pillar cell and the tunnel of Corti. Ribbon synapses precisely located at the plane of section of IHCs were not assigned a specific allocation.

### Statistical analyses

Data were summarized across all age groups and within each age group using mean ± standard deviation (SD). The number of efferent MOC-OHC and afferent IHC-SGN synapses was modelled using linear mixed-effects models to assess differences between the age groups and different turns. A random intercept was included for each animal accounting for correlation between measurements from the same animal. To address heteroscedasticity when modeling the number of afferent IHC-SGN synapses, we allowed different variances for each combination of age group and cochlear turn. Contrasts were reported with 95% confidence intervals and associated *p*-value. The significance level was set to α = 5% for all statistical tests. Due to the exploratory nature of this study, no adjustment for multiple testing was applied. All statistical analyses were performed using the statistic open-source software R 3.6.2 (R Core Team 2020). For ordinary linear mixed-effects models, R-packages lme4 v1.1.27.1 (Bates et al., 2015) and lmerTest v3.1.3 (Kuznetsova et al., 2017) were utilized. The R-package nlme v3.1–153 was used for linear mixed-effects models accounting for heteroscedasticity (Pinheiro et al., 2021). The R-package ggstatsplot v0.8.0 was used for creating graphical representations (Patil, 2021). Figures were edited with Inkscape v1.3.2 open-source software.

## Results

### The synapse density of efferent MOC-OHC synapses remains highest in the cochlear middle turn

**Figure 1:**
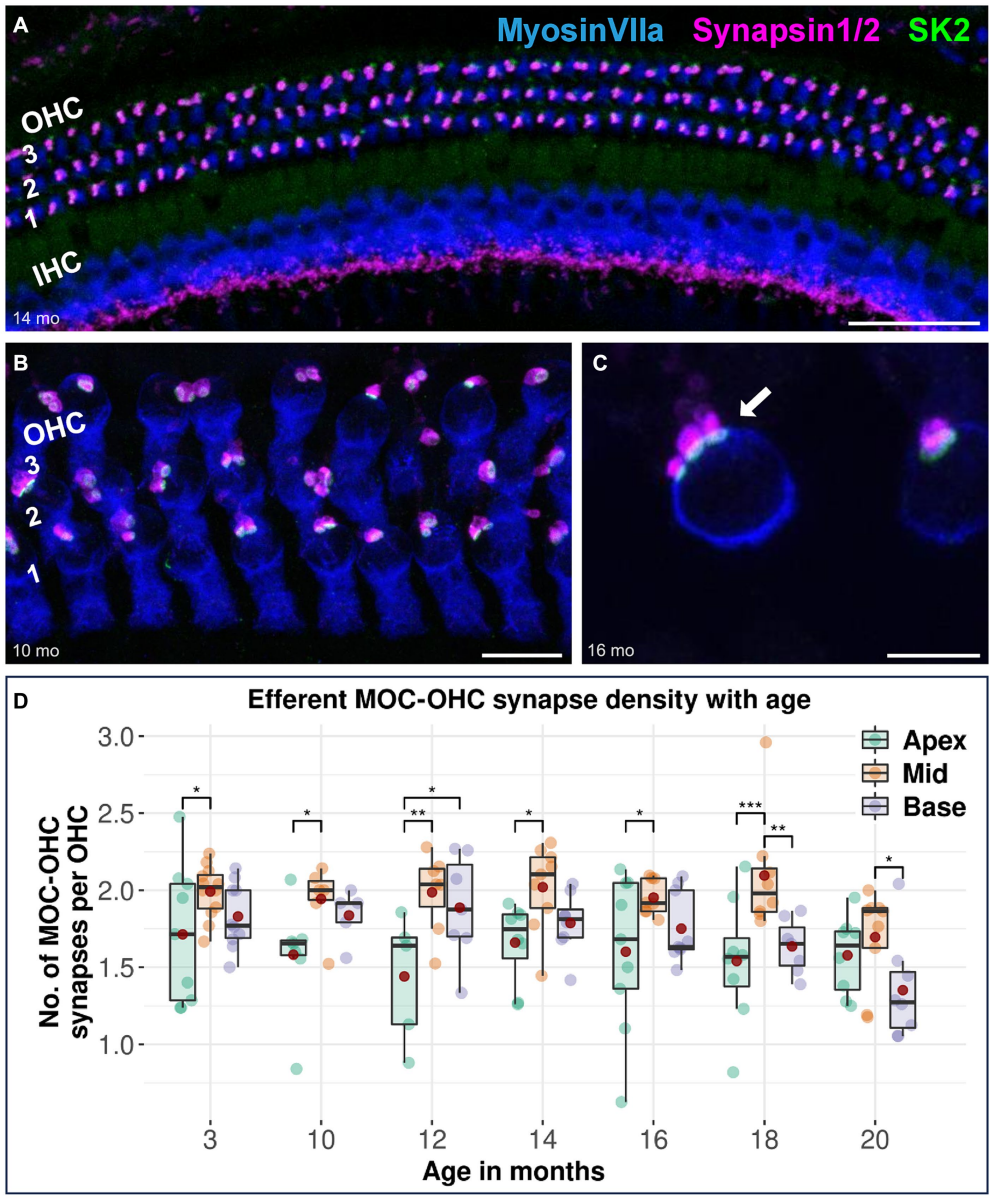
Confocal immunofluorescence analysis on efferent MOC-OHC synapses in CBA/J mice at different age stages. Representative confocal z-stack maximum projection images of organs of Corti’s middle turns from **(A)** 14-month-old, **(B)** 10-month-old, and **(C)** 16-month-old CBA/J mice. Whole mount organs of Corti were triple stained for hair cells (MyosinVIIa; blue), presynaptic MOC efferent nerve terminals (Synapsin1/2; magenta) and postsynaptic small-conductance Ca^2+^-activated K^+^ subtype 2 channels (SK2; green). The three OHC rows are numbered from 1 to 3, with the innermost OHC row corresponding to the first row. The white arrow points to a MOC-OHC efferent synapse showing juxtaposing presynaptic (Synapsin1/2) and postsynaptic (SK2) protein staining. MOC-OHC synapses displaying direct overlap between pre-and postsynaptic staining owing to spatial alignment appeared as white. Scale bar: **(A)** 40 μm, **(B)** 10 μm, **(C)** 5 μm. **(D)** Boxplots representing the number of intact efferent MOC-OHC synapses per OHC for apical, middle and basal cochlear turns at different age stages across the lifespan of CBA/J mice. Red dots represent the mean values. For each age group, intact efferent MOC-OHC synapses were compared between apical, middle, and basal turns using linear mixed-effects models, *p < 0.05, **p < 0.01, ***p < 0.001.

Efferent MOC-OHC synapses between MOC efferent nerve terminals and juxtaposed SK2 puncta within the basolateral membrane of OHCs remain most prominent in the cochlear middle turn across all age stages in CBA/J mice, with the middle turn exhibiting a significantly higher number of MOC-OHC synapses per OHC compared to the cochlear apex until 18 months of age (Figure 1). There were no discernible distinctions between male and female mice (data not shown). The formal comparison of efferent MOC-OHC synapses per OHC for each age group among cochlear regions is presented in Table 2.

**Table 2:** Comparison between cochlear regions at different age stages using a linear mixed-effects model for intact efferent MOC-OHC synapses per OHC.

**Age group**	**Contrast**	**Estimate**	**95%CI**	***p-*value**
3 months	apex – mid	−0.28	[−0.54, −0.02]	*0.037*
	base – mid	−0.16	[−0.41, 0.09]	0.212
	base – apex	0.12	[−0.15, 0.39]	0.396
10 months	apex – mid	−0.36	[−0.69, -0.04]	*0.032*
	base – mid	−0.12	[−0.47, 0.24]	0.527
	base – apex	0.25	[−0.1, 0.59]	0.162
12 months	apex – mid	−0.55	[−0.89, -0.2]	*0.002*
	base – mid	−0.1	[−0.41, 0.22]	0.552
	base – apex	0.45	[0.11, 0.8]	*0.011*
14 months	apex – mid	−0.36	[−0.64, -0.07]	*0.016*
	base – mid	−0.24	[−0.52, 0.05]	0.108
	base – apex	0.12	[−0.17, 0.42]	0.416
16 months	apex – mid	−0.35	[−0.64, -0.07]	*0.017*
	base – mid	–0.2	[−0.49, 0.08]	0.172
	base – apex	0.15	[−0.12, 0.43]	0.284
18 months	apex – mid	−0.56	[−0.85, -0.26]	*< 0.001*
	base – mid	−0.46	[−0.77, -0.16]	*0.004*
	base – apex	0.09	[−0.21, 0.4]	0.543
20 months	apex – mid	−0.12	[−0.4, 0.17]	0.419
	base – mid	−0.34	[−0.63, -0.06]	*0.020*
	base – apex	−0.23	[−0.52, 0.07]	0.134

### The cochlear apex consistently exhibits the lowest density of afferent IHC-SGN synapses

**Figure 2:**
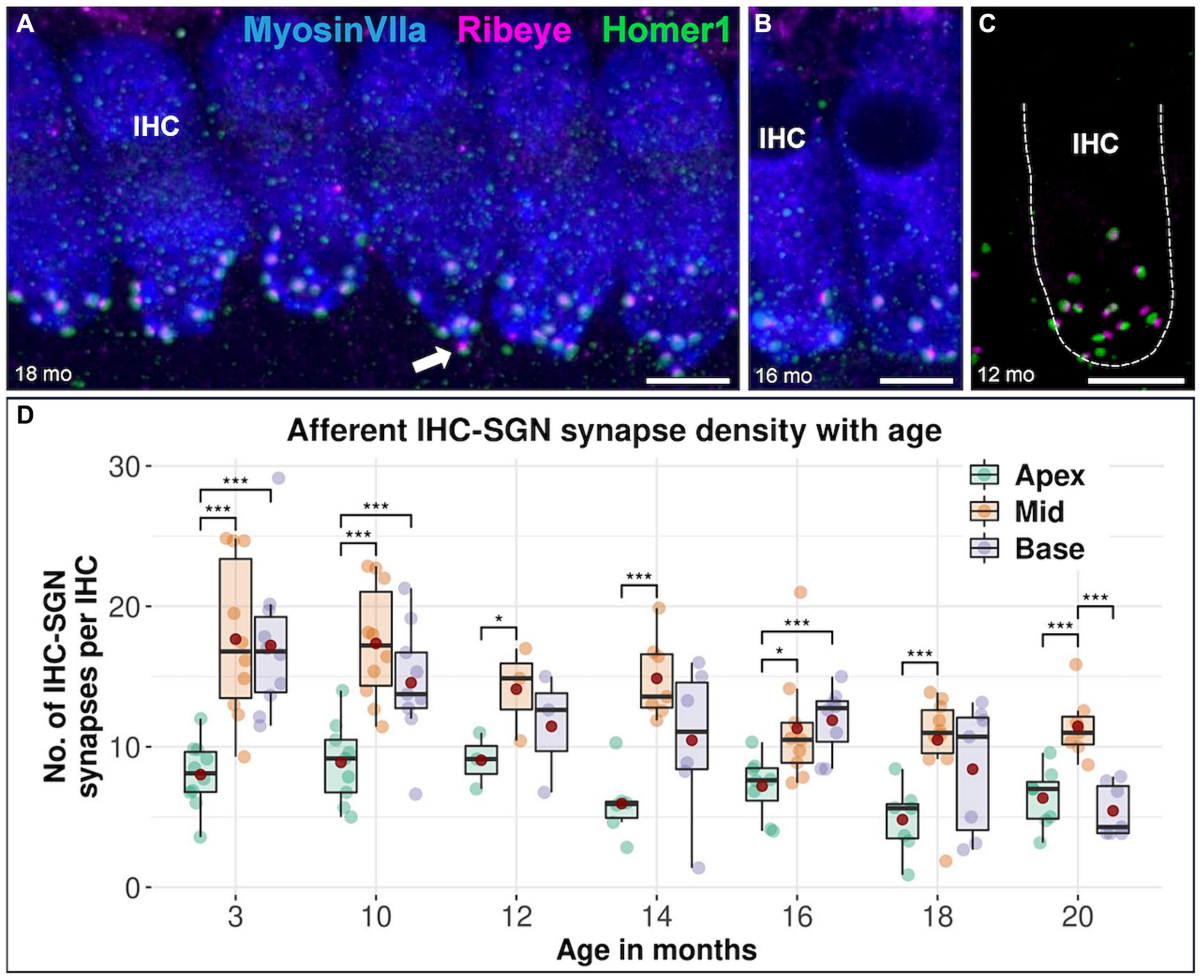
Confocal immunofluorescence analysis on afferent IHC-SGN ribbon synapses in CBA/J mice at different age stages. Representative confocal z-stack maximum projection images of organs of Corti’s apical IHC region from **(A)** 18-month-old, **(B)** 16-month-old, and **(C)** 12-month-old CBA/J mice. Whole mount organs of Corti were triple stained for hair cells (MyosinVIIa; blue), presynaptic ribbon (Ribeye A-Domain; magenta) and postsynaptic afferent type I SGN nerve terminal bouton (Homer1; green). The dashed white outline represents the plasma membrane’s schematic border of an individual IHC, intentionally excluding MyosinVIIa (blue) stain depiction to enhance the clarity of the synapse. Scale bar: 5 μm. The white arrows point to intact IHC-SGN afferent synapses showing juxtaposing presynaptic ribbon and postsynaptic type I SGN afferent bouton staining. **(D)** Boxplots representing the number of intact afferent IHC-SGN synapses per IHC for apical, middle and basal cochlear turns at different age stages across the lifespan of CBA/J mice. Red dots represent the mean values. For each age group, intact afferent IHC-SGN synapses were compared between apical, middle, and basal turns using linear mixed-effects models, *p < 0.05, **p < 0.01, ***p < 0.001.

The synapse density of afferent IHC-SGN ribbon synapses in the apical turn is consistently lower than that in the middle turn across all age groups, including in the youngest 3-month-old animals. Notably, significant differences in synapse density between the apical turn and the cochlear base are observed at 3, 10, and 16 months of age. When comparing the cochlear base with the middle turn in each specific age group, only the oldest group of mice aged 20 months exhibited significantly lower IHC-SGN synapse density on the base compared to the middle turn (Figure 2). There were no discernible distinctions between male and female mice (data not shown). The formal comparison of afferent IHC-SGN ribbon synapses per IHC for each age group among cochlear regions, is presented in Table 3.

**Table 3:** Comparison of cochlear regions at different age stages using a linear mixed-effects model for intact afferent IHC-SGN ribbon synapses per IHC.

**Age group**	**Contrast**	**Estimate**	**95%CI**	***p-*value**
3 months	apex – mid	−9.79	[−12.93, −6.66]	*<0.001*
	base – mid	−0.58	[−5.07, 3.91]	0.797
	apex – base	−9.21	[−12.65, −5.77]	*<0.001*
10 months	apex – mid	−8.43	[−10.68, −6.18]	*<0.001*
	base – mid	−2.84	[−6.05, 0.36]	0.082
	apex – base	−5.59	[−8.39, −2.79]	*<0.001*
12 months	apex – mid	−3.82	[−7.17, −0.47]	*0.026*
	base – mid	−1.40	[−5.68, 2.88]	0.516
	apex – base	−2.42	[−5.09, 0.25]	0.075
14 months	apex – mid	−8.70	[−10.26, −7.14]	<0.001
	base – mid	−4.51	[−9.23, 0.2]	0.060
	apex – base	−4.18	[−8.83, 0.46]	0.077
16 months	apex – mid	−4.08	[−7.48, −0.67]	*0.020*
	base – mid	0.66	[−2.64, 3.95]	0.693
	apex – base	−4.73	[−6.97, −2.5]	*<0.001*
18 months	apex – mid	−5.50	[−7.54, −3.46]	*<0.001*
	base – mid	−2.38	[−6.09, 1.34]	0.206
	apex – base	−3.12	[−6.59, 0.36]	0.078
20 months	apex – mid	−5.23	[−6.87, −3.59]	*<0.001*
	base – mid	−5.79	[−7.27, −4.31]	*<0.001*
	apex – base	0.56	[4.31, 7.27]	*<0.001*

### Efferent MOC-OHC synapses diminish uniformly across all three OHC rows in aging CBA/J mice, reaching a significant decline at 20 months

**Figure 3:**
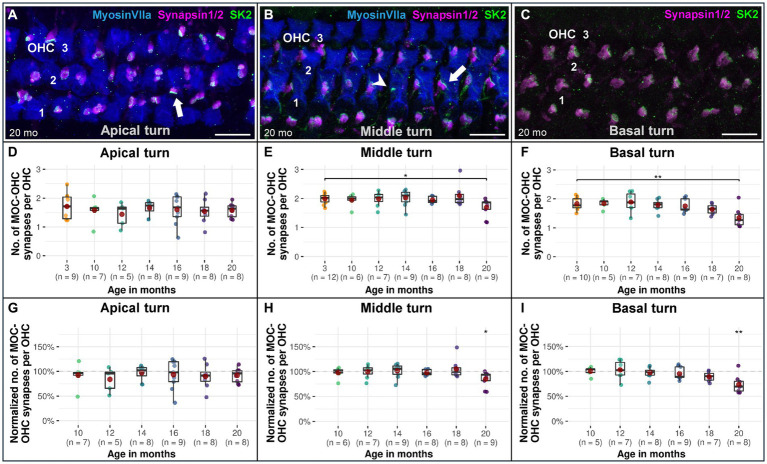
Confocal immunofluorescence analysis on age-associated reduction of efferent MOC-OHC synapses in CBA/J mice. Representative confocal z-stack maximum projection images from organs of Corti’s OHC region corresponding to **(A)** 20-month-old apical turn, **(B)** 20-month-old middle turn, and **(C)** 20-month-old basal turn. Whole mount organs of Corti were triple stained for hair cells (MyosinVIIa; blue), presynaptic MOC efferent nerve terminals (Synapsin1/2; magenta) and postsynaptic small-conductance Ca^2+^-activated K^+^ subtype 2 channels (SK2; green). MOC-OHC synapses displaying direct overlap between pre-and postsynaptic staining owing to spatial alignment appeared as white. The MyosinVIIa (blue) stain depiction was intentionally excluded in panel C for better visualization of the synapses. Scale bar: 10 μm. The white arrows point to intact MOC-OHC efferent synapses showing juxtaposing presynaptic (Synapsin1/2) and postsynaptic (SK2) protein staining. The arrowhead points to a postsynaptic SK2 channel within the cell membrane of an OHC with an absent presynaptic MOC efferent nerve ending. Boxplots representing the number and the normalized number of intact efferent MOC-OHC synapses per OHC for the **(D,G)** apical turns, **(E,H)** middle turns, and **(F,I)** basal cochlear turns at different age stages of CBA/J mice. For each cochlear turn, data obtained from 10-, 12-, 14-, 16-, 18-, and 20-month-old animals were normalized by dividing the number of intact efferent MOC-OHC synapses by the corresponding mean of intact efferent MOC-OHC synapses obtained from the 3-month-old group, i.e., 100% corresponds to the youngest age group. Red dots represent the mean values. All age groups were compared to the youngest age group using linear mixed-effects models, *p < 0.05, **p < 0.01, ***p < 0.001.

**Figure 4:**
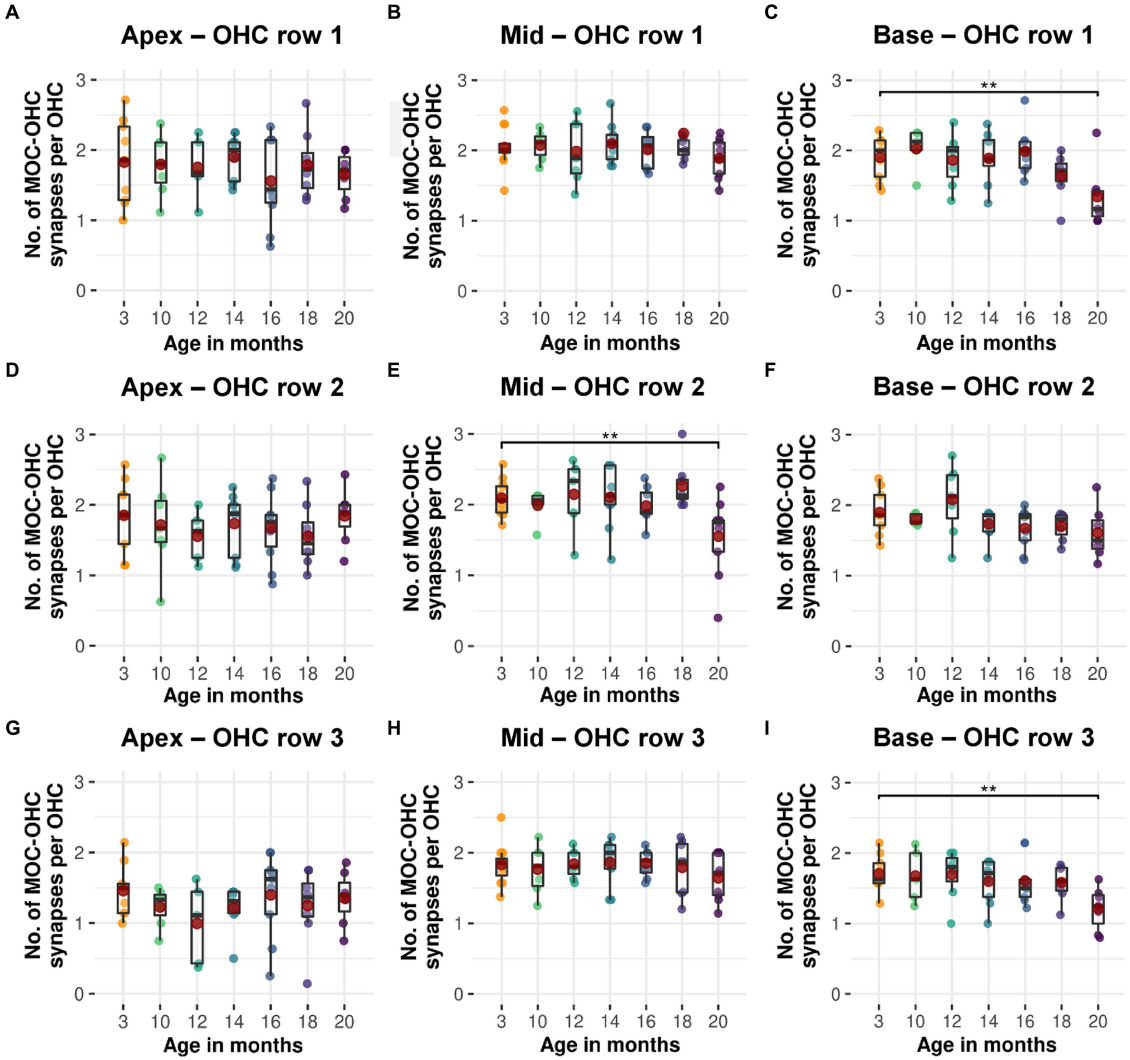
Age-related decline in efferent MOC-OHC synapses for each of the three OHC rows in CBA/J mice. Boxplots of intact efferent MOC-OHC synapses per OHC at different age stages of CBA/J mice corresponding to **(A–C)** OHC row 1, **(D–F)** OHC row 2, and **(G–I)** OHC row 3 for **(A,D,G)** apical turn, **(B,E,H)** middle turn, and **(C,F,I)** basal turn. Red dots represent the mean values. All age groups were compared to the youngest age group using linear mixed-effects models, *p < 0.05, **p < 0.01, ***p < 0.001.

Efferent MOC-OHC synapses in CBA/J mice exhibit a significant loss due to aging at the basal and middle turns of the cochlea by 20 months of age (Figure 3). Regarding the number of efferent MOC-OHC synapses within the 3-rows-subdivision of OHCs within the organ Corti, OHC row 1 and 3 show a significant decrease of efferent synapses in the cochlear base at 20 months of age when compared to 3-month-old littermates. OHC row 2 was indicative of a significant age-related reduction of efferent MOC-OHC synapses affecting the middle cochlear turn in mice aged 20 months (Figure 4). There were no discernible distinctions between male and female mice (data not shown). The formal statistical analysis for age-related changes is presented in Table 4. The depiction of data on MOC-OHC synapses per OHC, organized by age and cochlear region, along with the statistical analysis comparing age groups for each row of OHCs, is presented in Supplementary Tables S1, S2, respectively.

**Table 4:** Comparison between cochlear regions from older versus the youngest group using a linear mixed-effects model for intact efferent MOC-OHC synapses per OHC.

**Age group**	**Region**	**Estimate**	**95%CI**	***p-*value**
10 months	apex	−0.13	[−0.43, 0.17]	0.395
v./3 months	mid	−0.05	[−0.35, 0.25]	0.759
	base	0	[−0.33, 0.33]	0.995
12 months	apex	−0.28	[−0.61, 0.05]	0.103
v./3 months	mid	−0.01	[−0.29, 0.28]	0.958
	base	0.06	[−0.24, 0.35]	0.700
14 months	apex	−0.05	[−0.34, 0.24]	0.733
v./3 months	mid	0.03	[−0.23, 0.29]	0.831
	base	−0.05	[−0.33, 0.24]	0.751
16 months	apex	−0.11	[−0.39, 0.17]	0.435
v./3 months	mid	−0.04	[−0.31, 0.23]	0.781
	base	−0.08	[−0.35, 0.2]	0.578
18 months	apex	−0.17	[−0.46, 0.12]	0.243
v./3 months	mid	0.1	[−0.17, 0.38]	0.455
	base	−0.2	[−0.49, 0.1]	0.192
20 months	apex	−0.14	[−0.43, 0.15]	0.360
v./3 months	mid	−0.3	[−0.56, −0.03]	*0.029*
	base	−0.48	[−0.76, −0.2]	*0.001*

### A significant age-related decrease in afferent IHC-SGN ribbon synapses starts in the cochlear basal turn at 14 months of age

**Figure 5:**
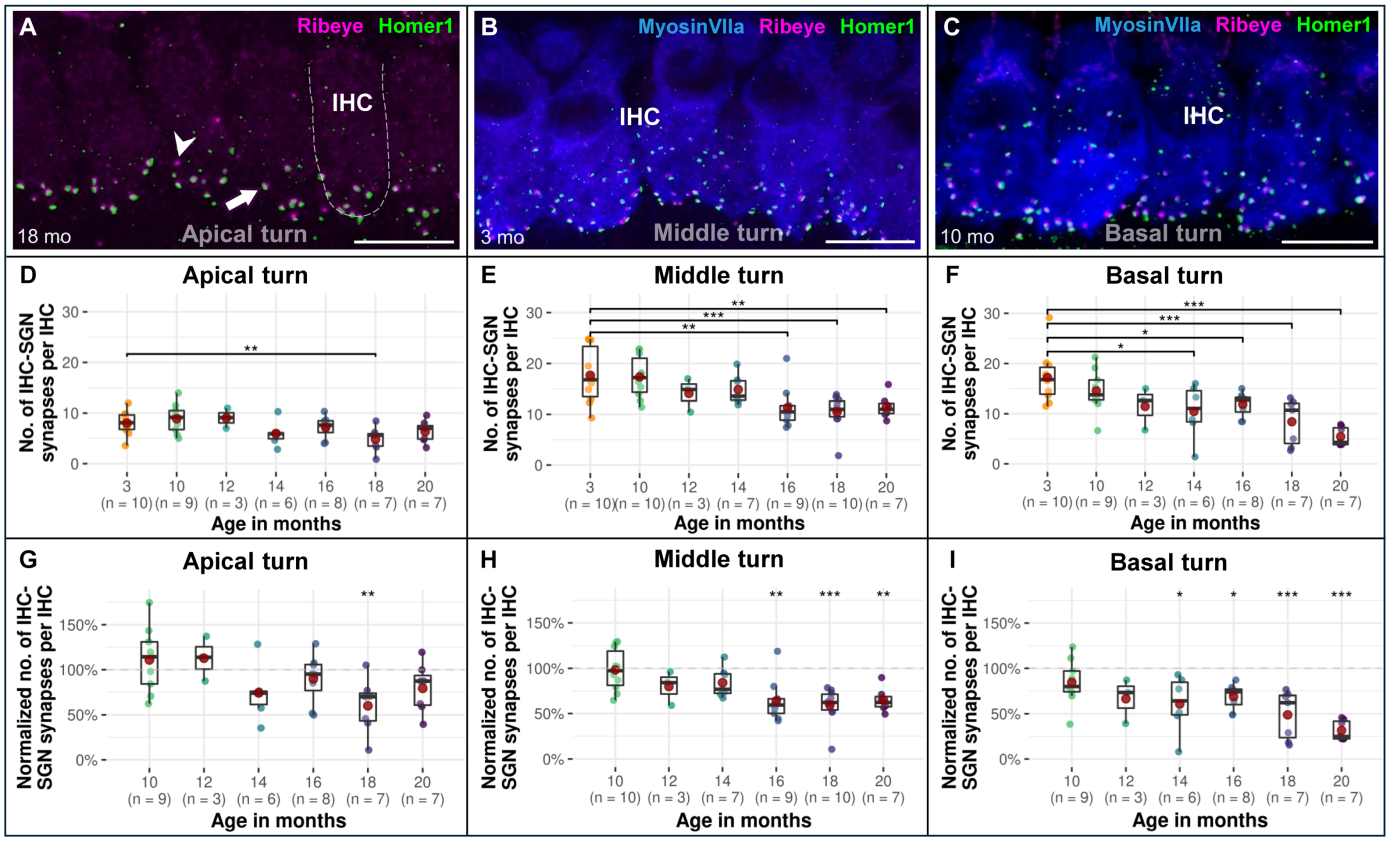
Confocal immunofluorescence analysis on age-associated decrease in afferent IHC-SGN ribbon synapses in CBA/J mice. Representative confocal z-stack maximum projection images from organs of Corti’s IHC row at different age stages corresponding to **(A)** 18-month-old apical turn, **(B)** 3-month-old middle turn, and **(C)** 10-month-old basal turn. Whole mount organs of Corti were triple stained for hair cells (MyosinVIIa; blue), presynaptic ribbon (Ribeye A-Domain; magenta) and postsynaptic afferent type I SGN nerve terminal bouton (Homer1; green). The dashed white outline represents the plasma membrane’s schematic border of an individual IHC, intentionally excluding MyosinVIIa (blue) stain depiction to enhance the clarity of the synapse. Scale bar: 10 μm. The white arrow points to an intact IHC-SGN afferent synapse showing juxtaposing presynaptic ribbon and postsynaptic type I SGN afferent bouton staining. The arrowhead points to an IHC ribbon with an absent postsynaptic counterpart. Boxplots representing the number and the normalized number of intact afferent IHC-SGN synapses per IHC for the **(D,G)** apical turns, **(E,H)** middle turns, and **(F,I)** basal cochlear turns at different age stages of CBA/J mice. For each cochlear turn, data obtained from 10-, 12-, 14-, 16-, 18-, and 20-month-old animals were normalized by dividing the number of intact afferent IHC-SGN synapses by the corresponding mean of intact afferent IHC-SGN synapses obtained from the 3-month-old group, i.e., 100% corresponds to the youngest age group. Red dots represent the mean values. All age groups were compared to the youngest age group using linear mixed-effects models, *p < 0.05, **p < 0.01, ***p < 0.001.

The decline of afferent IHC-SGN ribbon synapses due to the aging process in the lifespan of CBA/J mice first affects the cochlear basal turn, beginning at 14 months compared to 3-month-old littermates, gradually persisting until reaching the oldest 20-month-old group (Figures 5F,I). The cochlear middle turn experienced a significant progressive decline commencing in mice aged 16 months (Figures 5E,H), while the cochlear apex exhibits a significant age-related decline of afferent IHC-SGN ribbon synapses at 18 months of age (Figures 5D,G). There were no discernible distinctions between male and female mice (data not shown). The formal statistical analysis for age-related changes between cochlear regions is presented in Table 5. The depiction of data on IHC-SGN ribbon synapses per IHC, organized by age and cochlear region, is presented in Supplementary Table S3.

**Table 5:** Comparison between cochlear regions from older versus the youngest group using a linear mixed-effects model for intact afferent IHC-SGN ribbon synapses per IHC.

**Age group**	**Region**	**Estimate**	**95%CI**	***p-*value**
10 months	apex	0.82	[−1.38, 3.02]	0.459
v./3 months	mid	−0.54	[−4.46, 3.39]	0.786
	base	−2.8	[−7.32, 1.73]	0.222
12 months	apex	1.47	[−1.11, 4.05]	0.260
v. / 3 months	mid	−4.5	[−9.4, 0.4]	0.071
	base	−5.32	[−10.24, −0.4]	*0.034*
14 months	apex	−1.82	[−4.07, 0.44]	0.113
v./3 months	mid	−2.91	[−6.67, 0.85]	0.127
	base	−6.84	[−12.79, −0.9]	*0.025*
16 months	apex	−0.72	[−3.25, 1.81]	0.573
v./3 months	mid	−6.44	[−10.99, −1.89]	*0.006*
	base	−5.2	[−9.2, −1.2]	*0.012*
18 months	apex	−3	[−5.15, −0.85]	*0.007*
v./3 months	mid	−7.29	[−11.17, −3.41]	*< 0.001*
	base	−9.09	[−14.06, −4.13]	*< 0.001*
20 months	apex	−1.75	[−4.06, 0.56]	0.136
v./3 months	mid	−6.31	[−9.99, −2.62]	*0.001*
	base	−11.52	[−15.42, −7.62]	*< 0.001*

### Modiolar and pillar afferent IHC-SGN ribbon synapses in the middle and basal turns show a comparable age-related decline

**Figure 6:**
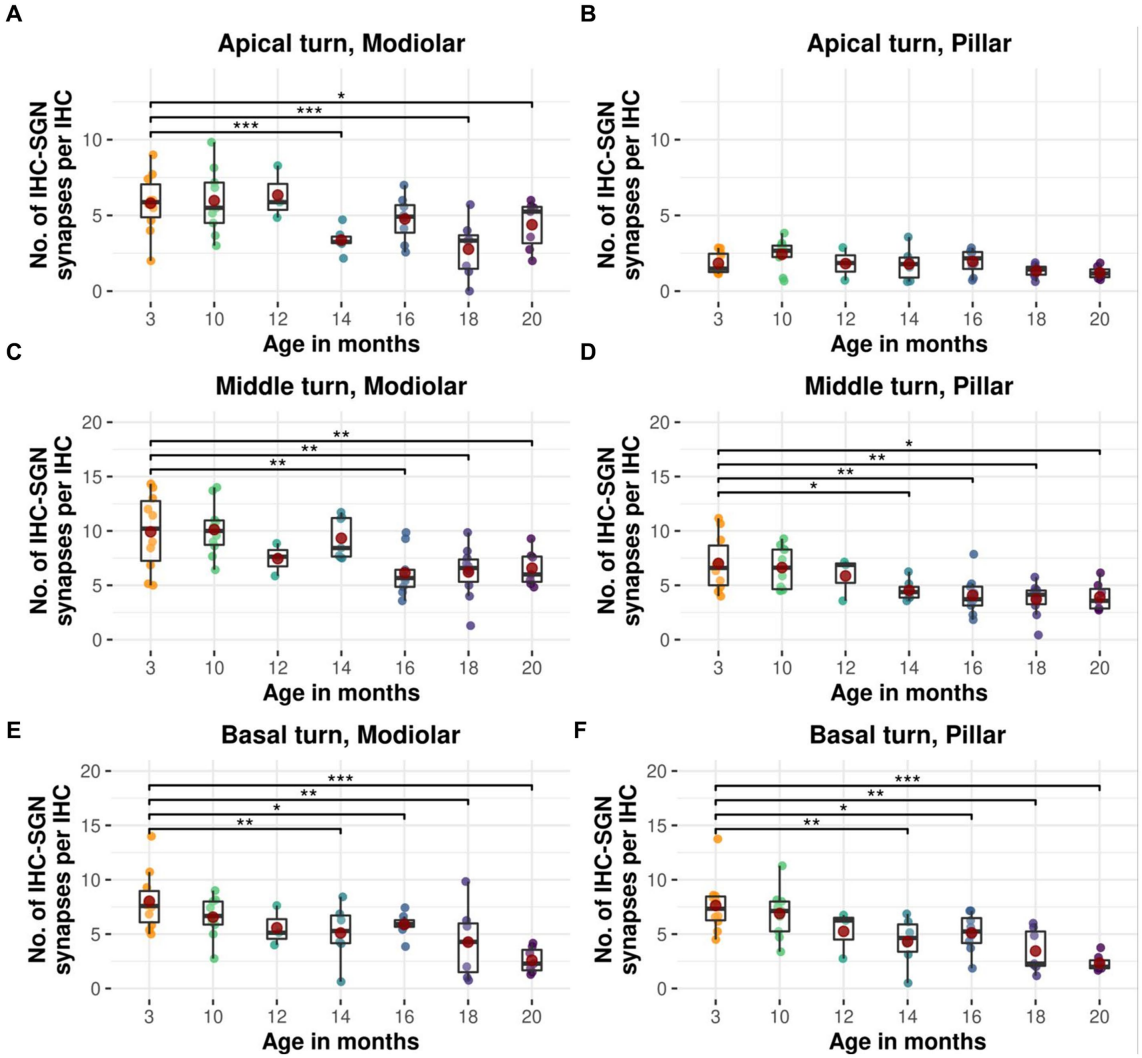
Age-related decline in modiolar and pillar afferent IHC-SGN ribbon synapses in CBA/J mice. Boxplots of intact modiolar afferent IHC-SGN synapses per IHC for cochlear **(A)** apical turn, **(C)** middle turn, and **(E)** basal turn, versus intact pillar afferent IHC-SGN synapses per IHC for **(B)** apical turn, **(D)** middle turn, and **(F)** basal turn at different age stages of CBA/J mice. Red dots represent the mean values. All age groups were compared to the youngest age group using linear mixed-effects models, *p < 0.05, **p < 0.01, ***p < 0.001.

Modiolar and pillar afferent IHC-SGN ribbon synapses from the middle and basal cochlear turns exhibit a comparable age-related reduction (Figure 6). Modiolar IHC-SGN ribbon synapses show a significant decline due to aging beginning at 14 months for the basal turn, and at 16 months of age for the middle turn (Figures 6C,E), whereas pillar IHC-SGN ribbon synapses demonstrate a significant age-related decline starting at 14 months of age, compromising both the middle and basal turns (Figures 6D,F). The cochlear apex shows a significant age-associated decline on modiolar ribbon synapses at 14, 18, and 20 months of age (Figure 6A), while pillar synapses exhibit no significant age-associated reduction when compared to 3-month-old littermates (Figure 6B). The depiction of data on modiolar and pillar IHC-SGN synapses per IHC, organized by age and cochlear region, as well as their formal statistical analysis for age-related changes is presented in Supplementary Tables S3, S4, respectively.

## Discussion

Our results on age-related cochlear findings in CBA/J mice have revealed the following insights: (1) Efferent MOC-OHC synapses exhibit the highest synaptic density in the middle turn of the cochlea across all age groups. (2) Efferent MOC-OHC synapses decrease across all three rows of OHCs, becoming significant in the middle and basal turns at 20 months. (3) The cochlear apex consistently demonstrates the lowest density of afferent IHC-SGN ribbon synapses. (4) The loss of integral synapses associated with myelinated auditory nerve fibers primarily affects afferent IHC-SGN ribbon synapses. (5) A similar pattern of decline in modiolar and pillar afferent IHC-SGN ribbon synapses is observed in the middle and basal turns, commencing at 16 and 14 months of age, respectively.

### Synapse density of MOC-OHC and IHC-SGN synapses along the cochlear spiral

In the OHC region, MOC efferent fibers contacting OHCs showed the highest density in the middle turn of the cochlea throughout the lifespan of CBA/J mice (Figure 1). This is consistent with previous findings in both the CBA/J (Park et al., 2017) and CBA/CaJ mouse strains (Maison et al., 2003). Our data regarding the first afferent auditory synapse between IHCs and type I afferent nerve fibers indicate the lowest number within the low-frequency region. It is noteworthy that the cochlear apex consistently showed the lowest density of afferent ribbon synapses regardless of age (Figure 2). The afferent synaptic counts assessed within the middle frequency region of our youngest age group, at 3 months of age, accounted for a mean of 16.8 IHC-SGN ribbon synapses per IHC. This is consistent with previous observations from the cochlear middle turn in 1-month-old CBA/CaJ mice, where an identical mean of 16.8 synapses per IHC was reported (Kujawa and Liberman, 2009), as well as in C57BL/6 J mice, with 16.3 ribbon synapses per IHC at 2 weeks of age (Peineau et al., 2021).

### Efferent MOC-OHC synapses uniformly diminish with age

A gradual and slow reduction of efferent MOC-OHC synapses across the three OHC rows was observed in CBA/J mice across their lifespan, with a significant decrease observed at 20 months when compared to 3-month-old littermates (Figures 3, 4). Previous research on CBA/CaJ mice have demonstrated an age-associated decrease in the number of MOC neurons within the brainstem (Vicencio-Jimenez et al., 2021), in addition to changes in OHC morphology in aging C3H/HeJ mice, were middle turn OHCs exhibited a significant decrease in surface area at 6 months of age compared to younger counterparts, yet without affecting OHCs’ function, determined by distortion product otoacoustic emissions (DPAOEs) (Jeng et al., 2020). The C3H/HeJ mouse strain, known for its similar physiological features to CBA/J mice, is also considered as a ‘good hearing’ mouse strain. Remarkably, at 15 months, C3H/HeJ mice did not show a significant age-related decline on middle turn efferent MOC-OHC synapses (Jeng et al., 2020), consistent with our observations. In humans, post-mortem studies have demonstrated a decrease in the efferent MOC projections to OHCs with age (Liberman and Liberman, 2019). Audiological assessments of the MOC efferent system have revealed a weakening of its function as individuals age, particularly in the high frequency range (Lisowska et al., 2014). The observed decline is indicated by a diminished inhibitory response in the contralateral suppression (CS) of DPOAEs. This reduction in CS of DPOAEs with age was measured when pure tones were applied to the contralateral ear, indirectly suggesting a functional efferent MOC deterioration (Varghese et al., 2005). The diminished activity of the MOC system appears to precede a decline of OHC function with age, as observed in both CBA mice (Jacobson et al., 2003; Zhu et al., 2007) and humans (Kim et al., 2002; Konomi et al., 2014). Based on our observations, it is plausible to hypothesize that a reduction in the number of MOC-OHCs synapses may contribute to the observed decline in the CS of DPOAEs with advancing age, particularly in the high frequency range. However, a synaptic decline does not inherently correlate with a quantifiable decrease in function, as the diminished inhibitory function of the MOC efferent system with age likely stems from multifactorial influences. Research indicates that OHC function in CBA mice declines later in their life, as demonstrated by a marked decrease in DPOAEs surpassing 20–25 dB at the age of 25 months and older (Parham et al., 1999; Parthasarathy and Kujawa, 2018). This onset of decline is noteworthy given that the median lifespan of the CBA/J mouse strain ranges from 22 to 25 months (Smith et al., 1973). Considering that cell functionality does not appear to be dictated by morphological changes associated with aging (Bortner and Cidlowski, 2003), it is possible that alterations in OHC function may only become apparent following OHC apoptosis. The evidence suggesting a decline in CS-DPAOE activity due to aging, which precedes deterioration in DPOAEs, indicates that MOC function may deteriorate before there is actual loss of OHCs. Supporting this notion, an early study on this mouse strain showed no hair cell loss along the cochlear spiral up to the age of 18 months (Spongr et al., 1997). In non-systematic observations, the detection of a single OHC missing in CBA/J mice, regardless of age or frequency range, was an exceedingly rare occurrence to observe. Although age-related loss of OHCs was not clearly evidenced in CBA/J mice in this study, it is possible that it may become evident later in their lifespan. However, since this study did not proactively track changes in OHC morphology or count, we refrain from drawing conclusive insights in this regard. It is noteworthy to mention that, despite the aging process, the arrangement of efferent synaptic connections between MOC fibers and OHCs remained morphologically stable throughout our analysis of MOC-OHC synapses along the cochlear spiral. This observation is particularly significant in light of reports on the formation of *de novo* efferent LOC synaptic contacts on aged IHCs in C57BL/6 mice (Lauer et al., 2012) resembling those that existed prior to the onset of hearing (Zachary and Fuchs, 2015).

### Afferent IHC synaptopathy precedes efferent MOC-OHC synaptic decline

The auditory synapses linked to myelinated nerve fibers in the organ of Corti of CBA/J mice show a decrease with age, predominantly impacting the afferent IHC-SGN ribbon synapses. This observation agrees with previous findings pointing to the first auditory synapse as the most vulnerable structure within the peripheral auditory system (Kujawa and Liberman, 2009; Sergeyenko et al., 2013; Parthasarathy and Kujawa, 2018). When monitoring the colabeling pattern between MOC efferent terminals and the postsynaptic SK2 channels on OHCs as mice age, our findings suggest that the loss of efferent MOC-OHC synapses occurs noticeably later compared to the afferent ribbon synapses. This form of efferent OHC synaptopathy exhibits a delay in morphological downturn of at least 6 months compared to synapses between IHCs and type I SGNs (Figures 3, 5). Given that a functional assessment of synaptic activity is absolutely necessary to draw any further conclusions on this matter, it is important to approach the observed changes in synaptic disengagement with caution. It also remains to be noted that we did not monitor age-related changes in the immunoreactivity of large conductance Ca^2+^-activated K^+^ (BK) channels on OHCs (Lee and Cui, 2010), known to play a specific role in the basal cochlear turn (Wersinger et al., 2010; Rohmann et al., 2015). To our knowledge, this study constitutes the first comprehensive analysis to concurrently examine the alterations in cochlear synapses related to myelinated auditory nerve fibers as mice age. Our findings concur with previous research by Sergeyenko et al. on afferent synaptic morphological integrity of IHCs, which shows that age is associated with the deterioration of ribbon synapses throughout the cochlear spiral in CBA/CaJ mice (Sergeyenko et al., 2013). However, our results indicate that deafferentation begins no later than 14 months of age and progressively continues following a base-to-apex gradient (Figures 2, 5). In contrast to our observations, the age-associated reduction in afferent IHC-SGN ribbon synapses observed by Sergeyenko et al. in CBA/CaJ mice was initially noted in the apical turn of the cochlea. Furthermore, their data indicated that ribbon synapses in the basal turn were comparatively more resilient, displaying a relatively higher survival rate than those in the middle and apical cochlear regions, up until the mice aged beyond 25 months (Sergeyenko et al., 2013).

### Age-related decline in afferent ribbon synapses occurs on modiolar and pillar sides

IHC-SGN ribbon synapses exhibit a characteristically wide dynamic range in response to sound stimuli (Moser et al., 2006), which is associated with specific sensitivity to sound pressure levels linked to type I SGNs with characteristic spontaneous firing rates (SRs). The level of SR is inversely correlated with threshold sensitivity (Winter et al., 1990). Based on threshold differences (Whitfield, 1967; Liberman, 1978), afferent IHC-SGN ribbon synapses positioned along the modiolar aspect of the basolateral membrane of IHCs primarily respond to acoustic stimuli of medium to high intensity and connect to SGNs with low to medium SRs. Synapses oriented towards the pillar side of IHCs are responsive to acoustic inputs of low sound pressure and are typically connected to SGNs with high SRs (Liberman, 1982; Liberman et al., 2011). In mice, a continuum is formed by the arrangement of SGNs with SRs spanning from low to high (Shrestha et al., 2018). Our findings indicate that in the basal cochlear region commencing from 14 months of age, and progressing to the cochlear middle turn at 16 months, afferent IHC-SGN ribbon synapses display a uniform and consistent degenerative pattern of decline on both the modiolar and pillar sides of IHCs, signaling a steadily progressive deterioration IHC-SGN synapses due to aging (Figure 6). This pattern of decline follows a base to apex gradient (Figure 5). However, we observed a conspicuous deterioration of ribbon synapses at the modiolar surface of IHCs in CBA/J mice within the apical turn in older age stages, leading to a pronounced decline on modiolar IHC-SGN synapses within the low frequency region. In a study by Peineau et al. on C57BL/6 J mice, a comparison of IHC-SGN ribbon synapses at 1 and 12 months of age showed a preferential loss of modiolar ribbon synapses in the group of older mice at 12 months (Peineau et al., 2021). In light of this observation, it is imperative that we approach any interpretation and extrapolation of age-related data from the inner ear of C57BL/6 J mice with care. This caution is due to the well-documented single G → A transition at nucleotide 753 in exon 7 of the gene encoding cadherin 23, which causes in-frame exon skipping and results in a mutant form of cadherin 23 (Noben-Trauth et al., 2003). This impacts the protein’s long-term ability to transmit force to mechanically gated ion channels (Siemens et al., 2004), consequently disturbing the physiology and expected fate of hair cells due to aging. Our findings, however, reveal a consistent pattern of IHC synaptopathy that is independent of modiolar or pillar orientation, showing a continuum of degradation from high to low frequencies with age. Selective cochlear synaptopathy has received considerable attention in recent decades in relation to noise trauma, with glutamate-induced excitotoxicity identified as one of the main contributors (Puel et al., 1994; Kurabi et al., 2017; Sebe et al., 2017; Hu et al., 2020). The relationship between noise overstimulation and the selective acoustic injury to modiolar IHC-SGN synapses and afferent nerve fibers has produced somewhat contradictory findings in CBA mice. Results vary, with studies showing either no impact on high-threshold SGNs (Suthakar and Liberman, 2021) or inconclusive effects (Liberman and Liberman, 2015). Recent research reveals a selective loss to high-threshold afferent nerve fibers, resulting in noise-induced injury to modiolar IHC-SGN synapses connecting to SGNs with low to medium SRs (Moverman et al., 2023). Consequently, our study on aging CBA/J mice has revealed a widespread and progressive loss of IHC-SGN ribbon synapses along the entire basolateral membrane of IHCs, impacting SGNs irrespective of their SR nature (Figures 5, 6).

In conclusion, our study reveals an age-related decrease in efferent synapse colabeling between MOC efferent terminals and SK2 channels on OHCs in CBA/J mice beginning in the base and middle turn of the cochlea at a relatively advanced stage within their lifespan. The decline of morphologically intact auditory synapses in the organ of Corti linked to myelinated auditory nerve fibers appears to first impact afferent ribbon synapses as mice age, with a loss of both modiolar and pillar IHC-SGN synapses following a base-to-apex gradient. While a functional assessment of synaptic activity remains crucial for drawing further conclusions, the observed pattern of age-related structural synaptic decline in CBA/J mice suggests that age-related afferent IHC synaptopathy begins significantly prior to the morphological downturn of efferent MOC-OHC synapses.

## Data availability statement

The original contributions presented in the study are included in the article/Supplementary material, further inquiries can be directed to the corresponding author/s.

## Ethics statement

The animal study was approved by LAVES - Niedersächsiches Landesamt für Verbraucherschutz und Lebensmittelsicherheit. The study was conducted in accordance with the local legislation and institutional requirements.

## Author contributions

ND: Writing – original draft. LS: Writing – original draft. FK: Writing – original draft. TO: Writing – original draft, Resources. NS: Writing – original draft, Resources. DB: Writing – original draft, Funding acquisition, Resources. CS: Writing – review & editing, Writing – original draft.
