# Supplementary material for: Age-related alterations in efferent medial olivocochlear-outer hair cell and primary auditory ribbon synapses in CBA/J mice
Source: Front Cell Neurosci. 2024 Jun 26;18:1412450. doi: 10.3389/fncel.2024.1412450 (PMC11234844; doi:10.3389/fncel.2024.1412450)
Supplement: Supplementary file 1 [file Data_Sheet_1.docx]

**Supplemental Table 1: Mean ± standard deviation of intact efferent MOC-OHC synapses per OHC for each age group and cochlear region**

| Age group | Region | | OHC row 1 | | | | | OHC row 2 | | | OHC row 3 | | OHC rows 1-3 |  |  |  |  |  |  |
| --- | --- | --- | --- | --- | --- | --- | --- | --- | --- | --- | --- | --- | --- | --- | --- | --- | --- | --- | --- |
| 3 months | | apex | | | 1.82 ± 0.61 | | 1.85 ± 0.52 | | | | 1.46 ± 0.37 | | 1.71 ± 0.45 | | | | | |  |
|  | | mid | | | 2.04 ± 0.29 | | 2.09 ± 0.25 | | | | 1.83 ± 0.28 | | 1.99 ± 0.17 | | | | | |  |
|  | | base | | | 1.90 ± 0.31 | | 1.90 ± 0.32 | | | | 1.71 ± 0.26 | | 1.83 ± 0.21 | | | | | |  |
| 10 months | | apex | | | 1.80 ± 0.44 | | 1.72 ± 0.64 | | | | 1.23 ± 0.27 | | 1.58 ± 0.37 | | | | | |  |
|  | | mid | | | 2.07 ± 0.22 | | 1.99 ± 0.21 | | | | 1.77 ± 0.37 | | 1.94 ± 0.22 | | | | | |  |
|  | | base | | | 2.02 ± 0.31 | | 1.80 ± 0.08 | | | | 1.68 ± 0.38 | | 1.84 ± 0.17 | | | | | |  |
| 12 months | | apex | | | 1.75 ± 0.45 | | 1.56 ± 0.36 | | | 0.997 ± 0.57 | | 1.44 ± 0.41 | | |  |  |  |  |  |
|  | | mid | | | 1.99 ± 0.45 | | 2.14 ± 0.48 | | | 1.84 ± 0.21 | | 1.99 ± 0.26 | | | |  |  |  |  |
|  | | base | | | 1.86 ± 0.38 | | 2.08 ± 0.50 | | | 1.69 ± 0.36 | | 1.89 ± 0.34 | | | |  |  |  |  |
| 14 months | | apex | | | 1.90 ± 0.33 | | 1.74 ± 0.44 | | | | 1.22 ± 0.32 | | 1.66 ± 0.26 | | | | | | |
|  | | mid | | 2.10 ± 0.29 | | 2.10 ± 0.45 | | | 1.86 ± 0.33 | | | 2.02 ± 0.28 | | | | | |  |  |
|  | | base | | 1.89 ± 0.37 | | 1.74 ± 0.21 | | | 1.60 ± 0.31 | | | 1.79 ± 0.20 | | | | | |  |  |
| 16 months | | apex | | 1.56 ± 0.61 | | 1.67 ± 0.49 | | | 1.40 ± 0.61 | | | 1.60 ± 0.51 | | | | | |  |  |
|  | | mid | | 2.01 ± 0.27 | | 1.98 ± 0.26 | | | 1.85 ± 0.20 | | | 1.95 ± 0.11 | | | | | |  |  |
|  | | base | | 1.99 ± 0.34 | | 1.67 ± 0.29 | | | 1.60 ± 0.33 | | | 1.75 ± 0.23 | | | | | |  |  |
| 18 months | | apex | | 1.79 ± 0.46 | | 1.55 ± 0.44 | | | 1.25 ± 0.52 | | | 1.54 ± 0.41 | | | | | |  |  |
|  | | mid | | 2.24 ± 0.63 | | 2.26 ± 0.33 | | | 1.79 ± 0.39 | | | 2.10 ± 0.38 | | | | | |  |  |
|  | | base | | 1.64 ± 0.32 | | 1.70 ± 0.20 | | | 1.58 ± 0.25 | | | 1.64 ± 0.18 | | | | | |  |  |
| 20 months | | apex | | 1.65 ± 0.33 | | 1.84 ± 0.39 | | | 1.35 ± 0.38 | | | 1.58 ± 0.25 | | | | | |  |  |
|  | | mid | | 1.89 ± 0.28 | | 1.55 ± 0.56 | | | 1.64 ± 0.32 | | | 1.70 ± 0.31 | | | | | |  |  |
|  | | base | | 1.34 ± 0.44 | | 1.61 ± 0.37 | | | 1.21 ± 0.31 | | | 1.35 ± 0.33 | | | | |  |  |  |

**Supplemental Table 2: Comparison between cochlear regions from older versus the youngest group for each row of OHCs using a linear mixed-effects model for intact efferent MOC-OHC synapses per OHC. The models were fitted separately for each region.**

| Region | OHC row | Contrast | Estimate | 95% CI | p value |
| --- | --- | --- | --- | --- | --- |
| apex | 1 | 10 months – 3 months | -0.03 | [-0.5, 0.44] | 0.902 |
| apex | 2 | 10 months – 3 months | -0.14 | [-0.61, 0.33] | 0.569 |
| apex | 3 | 10 months – 3 months | -0.23 | [-0.7, 0.24] | 0.335 |
| apex | 1 | 12 months – 3 months | -0.07 | [-0.59, 0.45] | 0.787 |
| apex | 2 | 12 months – 3 months | -0.3 | [-0.82, 0.22] | 0.265 |
| apex | 3 | 12 months – 3 months | -0.47 | [-0.98, 0.05] | 0.083 |
| apex | 1 | 14 months – 3 months | 0.07 | [-0.36, 0.51] | 0.739 |
| apex | 2 | 14 months – 3 months | -0.12 | [-0.56, 0.32] | 0.600 |
| apex | 3 | 14 months – 3 months | -0.29 | [-0.74, 0.15] | 0.203 |
| apex | 1 | 16 months – 3 months | -0.27 | [-0.7, 0.16] | 0.223 |
| apex | 2 | 16 months – 3 months | -0.18 | [-0.61, 0.24] | 0.403 |
| apex | 3 | 16 months – 3 months | -0.13 | [-0.56, 0.3] | 0.560 |
| apex | 1 | 18 months – 3 months | -0.04 | [-0.49, 0.42] | 0.874 |
| apex | 2 | 18 months – 3 months | -0.3 | [-0.75, 0.15] | 0.200 |
| apex | 3 | 18 months – 3 months | -0.21 | [-0.66, 0.24] | 0.361 |
| apex | 1 | 20 months – 3 months | -0.17 | [-0.64, 0.3] | 0.473 |
| apex | 2 | 20 months – 3 months | -0.01 | [-0.48, 0.46] | 0.969 |
| apex | 3 | 20 months – 3 months | -0.11 | [-0.58, 0.36] | 0.655 |
|  |  |  |  |  |  |
| mid | 1 | 10 months – 3 months | 0.03 | [-0.32, 0.38] | 0.869 |
| mid | 2 | 10 months – 3 months | -0.1 | [-0.45, 0.25] | 0.570 |
| mid | 3 | 10 months – 3 months | -0.07 | [-0.41, 0.28] | 0.708 |
| mid | 1 | 12 months – 3 months | -0.05 | [-0.38, 0.28] | 0.752 |
| mid | 2 | 12 months – 3 months | 0.05 | [-0.28, 0.38] | 0.755 |
| mid | 3 | 12 months – 3 months | 0.01 | [-0.32, 0.34] | 0.951 |
| mid | 1 | 14 months – 3 months | 0.06 | [-0.25, 0.36] | 0.721 |
| mid | 2 | 14 months – 3 months | 0.01 | [-0.29, 0.32] | 0.940 |
| mid | 3 | 14 months – 3 months | 0.03 | [-0.27, 0.34] | 0.838 |
| mid | 1 | 16 months – 3 months | -0.03 | [-0.35, 0.29] | 0.854 |
| mid | 2 | 16 months – 3 months | -0.11 | [-0.43, 0.21] | 0.494 |
| mid | 3 | 16 months – 3 months | 0.02 | [-0.29, 0.34] | 0.891 |
| mid | 1 | 18 months – 3 months | 0.2 | [-0.11, 0.52] | 0.212 |
| mid | 2 | 18 months – 3 months | 0.17 | [-0.15, 0.49] | 0.291 |
| mid | 3 | 18 months – 3 months | -0.04 | [-0.36, 0.27] | 0.795 |
| mid | 1 | 20 months – 3 months | -0.15 | [-0.46, 0.15] | 0.323 |
| mid | 2 | 20 months – 3 months | -0.54 | [-0.85, -0.24] | 0.001 |
| mid | 3 | 20 months – 3 months | -0.19 | [-0.49, 0.12] | 0.225 |

| base | 1 | 10 months – 3 months | 0.12 | [-0.23, 0.48] | 0.498 |
| --- | --- | --- | --- | --- | --- |
| base | 2 | 10 months – 3 months | -0.1 | [-0.46, 0.25] | 0.573 |
| base | 3 | 10 months – 3 months | -0.03 | [-0.39, 0.32] | 0.855 |
| base | 1 | 12 months – 3 months | -0.04 | [-0.36, 0.28] | 0.816 |
| base | 2 | 12 months – 3 months | 0.18 | [-0.14, 0.5] | 0.280 |
| base | 3 | 12 months – 3 months | -0.02 | [-0.34, 0.31] | 0.927 |
| base | 1 | 14 months – 3 months | -0.05 | [-0.36, 0.26] | 0.735 |
| base | 2 | 14 months – 3 months | -0.16 | [-0.47, 0.14] | 0.293 |
| base | 3 | 14 months – 3 months | -0.11 | [-0.41, 0.19] | 0.473 |
| base | 1 | 16 months – 3 months | 0.09 | [-0.22, 0.39] | 0.574 |
| base | 2 | 16 months – 3 months | -0.23 | [-0.53, 0.08] | 0.145 |
| base | 3 | 16 months – 3 months | -0.11 | [-0.41, 0.2] | 0.495 |
| base | 1 | 18 months – 3 months | -0.26 | [-0.59, 0.06] | 0.114 |
| base | 2 | 18 months – 3 months | -0.19 | [-0.52, 0.13] | 0.241 |
| base | 3 | 18 months – 3 months | -0.13 | [-0.45, 0.2] | 0.441 |
| base | 1 | 20 months – 3 months | -0.56 | [-0.88, -0.24] | 0.001 |
| base | 2 | 20 months – 3 months | -0.29 | [-0.61, 0.03] | 0.080 |
| base | 3 | 20 months – 3 months | -0.5 | [-0.82, -0.17] | 0.003 |

**Supplemental Table 3: Mean ± standard deviation of intact afferent IHC-SGN ribbon synapses per IHC for each age group and cochlear region**

| Age group | | Region | | Modiolar | Pillar | | Modiolar & Pillar | |  |  |
| --- | --- | --- | --- | --- | --- | --- | --- | --- | --- | --- |
| 3 months | apex | | 5.80 ± 1.99 | | | 1.84 ± 0.72 | 8.02 ± 2.4 |  |  |  |
|  | mid | | 9.92 ± 3.52 | | | 7.00 ± 2.55 | 17.7 ± 5.6 |  |  |  |
|  | base | | 8.02 ± 2.74 | | | 7.63 ± 2.57 | 17.2 ± 5.1 |  |  |  |
| 10 months | apex | | 5.98 ± 2.21 | | | 2.43 ± 1.05 | 8.89 ± 2.9 |  |  |  |
|  | mid | | 10.1 ± 2.4 | | | 6.63 ± 1.95 | 17.4 ± 4.1 |  |  |  |
|  | base | | 6.55 ± 1.90 | | | 6.89 ± 2.30 | 14.6 ± 4.3 |  |  |  |
| 12 months | apex | | 6.34 ± 1.76 | | | 1.82 ± 1.08 | 9.04 ± 2.0 |  |  |  |
|  | mid | | 7.45 ± 1.51 | | | 5.86 ± 1.99 | 14.1 ± 3.4 |  |  |  |
|  | base | | 5.58 ± 1.86 | | | 5.25 ± 2.18 | 11.5 ± 4.2 |  |  |  |
| 14 months | apex | | 3.37 ± 0.83 | | | 1.79 ± 1.11 | 5.95 ± 2.5 |  |  |  |
|  | mid | | 9.33 ± 1.93 | | | 4.52 ± 0.93 | 14.9 ± 2.9 |  |  |  |
|  | base | | 5.10 ± 2.73 | | | 4.32 ± 2.30 | 10.5 ± 5.5 |  |  |  |
| 16 months | apex | | 4.76 ± 1.49 | | | 1.95 ± 0.83 | 7.20 ± 2.2 |  |  |  |
|  | mid | | 6.14 ± 2.12 | | | 4.08 ± 1.79 | 11.3 ± 4.2 |  |  |  |
|  | base | | 5.89 ± 1.03 | | | 5.12 ± 1.81 | 11.9 ± 2.4 |  |  |  |
| 18 months | apex | | 2.77 ± 1.91 | | | 1.33 ± 0.43 | 4.82 ± 2.4 |  |  |  |
|  | mid | | 6.22 ± 2.36 | | | 3.72 ± 1.48 | 10.5 ± 3.4 |  |  |  |
|  | base | | 4.26 ± 3.30 | | | 3.44 ± 1.98 | 8.40 ± 4.6 |  |  |  |
| 20 months | apex | | 4.39 ± 1.59 | | | 1.21 ± 0.41 | 6.36 ± 2.2 |  |  |  |
|  | mid | | 6.58 ± 1.64 | | | 3.93 ± 1.30 | 11.5 ± 2.3 |  |  |  |
|  | base | | 2.60 ± 1.16 | | | 2.33 ± 0.74 | 5.44 ± 1.9 | | |  |

**Supplemental Table 4: Comparison between cochlear regions from older versus the youngest group using a linear mixed-effects model for intact modiolar and pillar afferent IHC-SGN ribbon synapses per IHC. The models were fitted separately for each region.**

| Region | Mod/Pill | Contrast | Estimate | 95% CI | p value |
| --- | --- | --- | --- | --- | --- |
| apex | modiolar | 10 months – 3 months | 0.17 | [-1.39, 1.74] | 0.824 |
| apex | pillar | 10 months – 3 months | 0.59 | [-0.97, 2.16] | 0.448 |
| apex | modiolar | 12 months – 3 months | 0.54 | [-1.45, 2.52] | 0.587 |
| apex | pillar | 12 months – 3 months | -0.02 | [-2.01, 1.96] | 0.982 |
| apex | modiolar | 14 months – 3 months | -2.43 | [-3.67, -1.19] | < 0.001 |
| apex | pillar | 14 months – 3 months | -0.05 | [-1.29, 1.19] | 0.933 |
| apex | modiolar | 16 months – 3 months | -1.04 | [-2.31, 0.23] | 0.106 |
| apex | pillar | 16 months – 3 months | 0.11 | [-1.15, 1.38] | 0.855 |
| apex | modiolar | 18 months – 3 months | -3.04 | [-4.49, -1.58] | < 0.001 |
| apex | pillar | 18 months – 3 months | -0.51 | [-1.96, 0.95] | 0.484 |
| apex | modiolar | 20 months – 3 months | -1.42 | [-2.8, -0.03] | 0.045 |
| apex | pillar | 20 months – 3 months | -0.63 | [-2.01, 0.76] | 0.365 |
|  |  |  |  |  |  |
| mid | modiolar | 10 months – 3 months | 0.21 | [-1.92, 2.34] | 0.843 |
| mid | pillar | 10 months – 3 months | -0.37 | [-2.5, 1.75] | 0.725 |
| mid | modiolar | 12 months – 3 months | -2.47 | [-4.96, 0.02] | 0.052 |
| mid | pillar | 12 months – 3 months | -1.14 | [-3.63, 1.35] | 0.361 |
| mid | modiolar | 14 months – 3 months | -0.59 | [-2.83, 1.65] | 0.598 |
| mid | pillar | 14 months – 3 months | -2.48 | [-4.72, -0.25] | 0.030 |
| mid | modiolar | 16 months – 3 months | -3.77 | [-5.93, -1.62] | 0.001 |
| mid | pillar | 16 months – 3 months | -2.92 | [-5.08, -0.76] | 0.009 |
| mid | modiolar | 18 months – 3 months | -3.69 | [-5.79, -1.6] | 0.001 |
| mid | pillar | 18 months – 3 months | -3.29 | [-5.39, -1.19] | 0.003 |
| mid | modiolar | 20 months – 3 months | -3.34 | [-5.66, -1.03] | 0.006 |
| mid | pillar | 20 months – 3 months | -3.08 | [-5.39, -0.76] | 0.010 |

| base | modiolar | 10 months – 3 months | -1.47 | [-3.44, 0.51] | 0.141 |
| --- | --- | --- | --- | --- | --- |
| base | pillar | 10 months – 3 months | -0.74 | [-2.72, 1.24] | 0.453 |
| base | modiolar | 12 months – 3 months | -2.44 | [-5.12, 0.24] | 0.073 |
| base | pillar | 12 months – 3 months | -2.38 | [-5.06, 0.3] | 0.080 |
| base | modiolar | 14 months – 3 months | -2.92 | [-5.04, -0.8] | 0.008 |
| base | pillar | 14 months – 3 months | -3.31 | [-5.43, -1.2] | 0.003 |
| base | modiolar | 16 months – 3 months | -2.13 | [-4.18, -0.09] | 0.042 |
| base | pillar | 16 months – 3 months | -2.52 | [-4.56, -0.47] | 0.017 |
| base | modiolar | 18 months – 3 months | -3.76 | [-6.26, -1.26] | 0.004 |
| base | pillar | 18 months – 3 months | -4.19 | [-6.69, -1.69] | 0.002 |
| base | modiolar | 20 months – 3 months | -5.42 | [-7.37, -3.48] | < 0.001 |
| base | pillar | 20 months – 3 months | -5.3 | [-7.25, -3.35] | < 0.001 |
