# Supplementary material for: Age-related alterations in efferent medial olivocochlear-outer hair cell and primary auditory ribbon synapses in CBA/J mice
Source: Front Cell Neurosci. 2024 Jun 26;18:1412450. doi: 10.3389/fncel.2024.1412450 (PMC11234844; doi:10.3389/fncel.2024.1412450)
Supplement: Supplementary file 2 [file Data_Sheet_2.docx]

**Table 5: Comparison between cochlear regions from older versus the youngest group using a linear mixed-effects model for intact afferent IHC-SGN ribbon synapses per IHC**

| Age group | | Region | | Estimate | | 95% CI | | p value | |
| --- | --- | --- | --- | --- | --- | --- | --- | --- | --- |
| 10 months | | apex | | | 0.82 | [-1.38, 3.02] | | | 0.459 |
| v. / 3 months | | mid | | | -0.54 | [-4.46, 3.39] | | | 0.786 |
|  | | base | | | -2.8 | [-7.32, 1.73] | | | 0.222 |
| 12 months | | apex | | | 1.47 | [-1.11, 4.05] | | | 0.260 |
| v. / 3 months | | mid | | | -4.5 | [-9.4, 0.4] | | | 0.071 |
|  | | base | | | -5.32 | [-10.24, -0.4] | | | 0.034 |
| 14 months | | apex | | | -1.82 | [-4.07, 0.44] | | | 0.113 |
| v. / 3 months | | mid | | | -2.91 | [-6.67, 0.85] | | | 0.127 |
|  | | base | | | -6.84 | [-12.79, -0.9] | | | 0.025 |
| 16 months | | apex | | | -0.72 | [-3.25, 1.81] | | | 0.573 |
| v. / 3 months | | mid | | | -6.44 | [-10.99, -1.89] | | | 0.006 |
|  | | base | | | -5.2 | [-9.2, -1.2] | | | 0.012 |
| 18 months | | apex | | | -3 | [-5.15, -0.85] | | | 0.007 |
| v. / 3 months | | mid | | | -7.29 | [-11.17, -3.41] | | | < 0.001 |
|  | | base | | | -9.09 | [-14.06, -4.13] | | | < 0.001 |
| 20 months | | apex | | | -1.75 | [-4.06, 0.56] | | | 0.136 |
| v. / 3 months | | mid | | | -6.31 | [-9.99, -2.62] | | | 0.001 |
|  | | base | | | -11.52 | [-15.42, -7.62] | | | < 0.001 |
|  |  | |  | | | |  |  | |
